# The Association between Monocyte Surface CD163 and Insulin Resistance in Patients with Type 2 Diabetes

**DOI:** 10.1155/2017/6549242

**Published:** 2017-12-28

**Authors:** Reina Kawarabayashi, Koka Motoyama, Miyuki Nakamura, Yuko Yamazaki, Tomoaki Morioka, Katsuhito Mori, Shinya Fukumoto, Yasuo Imanishi, Atsushi Shioi, Tetsuo Shoji, Masanori Emoto, Masaaki Inaba

**Affiliations:** ^1^Metabolism, Endocrinology, and Molecular Medicine, Osaka City University Graduate School of Medicine, Osaka, Japan; ^2^Department of Premier Preventive Medicine, Osaka City University Graduate School of Medicine, Osaka, Japan; ^3^Department of Vascular Medicine, Osaka City University Graduate School of Medicine, Osaka, Japan

## Abstract

**Aim:**

To investigate the association between monocyte CD163 and insulin resistance in patients with type 2 diabetes.

**Methods:**

One hundred sixty-six patients with type 2 diabetes without inflammatory or chronic kidney disease were recruited. The monocyte CD163 levels were measured by flow cytometry and soluble CD163 (sCD163) by ELISA. Insulin resistance was evaluated by the index of the homeostasis model assessment (HOMA-R).

**Results:**

The median sCD163 and monocyte CD163 expression levels were 582.9 (472.4–720.0) ng/ml and 6061 (4486–7876) mean fluorescent intensity (MFI), respectively. In a simple regression analysis, monocyte CD163 was inversely correlated with log [HOMA-R] (*r* = –0.257, *p* = 0.010), and sCD163 was positively correlated with log [HOMA-R] (*r* = 0.198, *p* = 0.042). In multiple regression analyses, monocyte CD163 was an independent contributor to log [HOMA-R] (*β* = –0.220, *p* = 0.020) even after adjustment of various clinical factors for HOMA-R (*R*^2^ = 0.281, *p* = 0.001), whereas sCD163 was not.

**Conclusions:**

Monocyte surface CD163 expression levels were more significantly associated with insulin resistance than sCD163 in patients with type 2 diabetes, suggesting a novel pathophysiological role of CD163.

## 1. Introduction

Cluster differentiation (CD) 163, a scavenger receptor for the haptoglobin-hemoglobin (Hb-Hp) complex specifically expressed on monocyte/macrophage lineages, has been extensively studied in hemoglobin-iron metabolism [1]. It belongs to the scavenger receptor cysteine-rich family, which is composed of a short transmembrane domain, a short cytoplasmic tail, and an extracellular domain [2]. The Hb-Hp complex (formed by the binding of hemoglobin to haptoglobin) in the circulation binds to monocyte surface CD163, which is subsequently incorporated by endocytosis and degenerated by heme oxygenase-1 (HO-1) to iron-containing heme, with reduced toxic properties [3, 4].

Recently, CD163 was also recognized to play a crucial role in various inflammatory diseases [2]. CD163 is cleaved from the surface of monocyte by metalloprotease tumor necrosis factor alpha (TNF*α*)-converting enzyme (TACE/ADAM17), and soluble form of CD163 (sCD163) is released into circulation in response to inflammatory stimuli [3]. In fact, the serum sCD163 level was found to increase in severe sepsis [5], liver failure [6], and chronic inflammatory diseases such as rheumatoid arthritis and cancer [2]. In some viral infections, such as HIV infections, serum sCD163 levels and CD163 expression levels on monocyte populations are recently known to be associated with clinical status and antiretroviral therapy [7-8]. Furthermore, sCD163 was also reported to be associated with insulin resistance, obesity, and atherosclerotic disease [9–13]. A large cohort study revealed that sCD163 was predictive of the onset of type 2 diabetes [14]. These findings suggest the possible use of sCD163 as a biomarker for insulin resistance and glucose intolerance, induced by inflammation.

On the other hand, CD163 expression on the surface of monocytes, a precursor of macrophages, has not been fully investigated, even though monocytes (as well as macrophages) have been recognized as a key player in chronic inflammation. Under steady-state conditions, the expression of CD163 on monocytes is 10- to 15-fold less than that of macrophages [15]. However, once an inflammatory stimulus is added, the response of monocyte CD163 has a similar trend to that of macrophages [15], which suggests that monocyte CD163 may be a useful marker for inflammation. There are few reports examining monocyte CD163 expression levels in diabetes. One report has demonstrated an increase in monocyte CD163 mRNA levels after very low calorie diet therapy (VLCD), with a decrease in sCD163 levels [16]. Another study found lower levels of monocyte CD163 expression in patients with type 2 diabetes having vascular complications than in those without vascular complications [17]. Furthermore, CD163 is actually expressed on monocyte cell surface especially in the CD14^high^CD16^+^ monocyte subpopulation that has been reported to exert predominantly anti-inflammatory functions [18, 19]. Most upregulators of CD163 expression on monocyte are anti-inflammatory cytokines such as IL-10 and glucocorticoids [20]. These evidences evoke us of a concept that monocyte CD163 is a potential protective marker for insulin resistance. To date, the association between monocyte CD163 and insulin resistance is not well studied. Therefore, it is necessary to elucidate the pathophysiological role and clinical implication of monocyte CD163 for insulin resistance.

In the present study, we investigated whether cell surface CD163 expression levels on peripheral blood monocytes (PBMs) as well as the level of sCD163 (in blood circulation) are associated with insulin resistance using homeostasis model assessment (HOMA-R) in a cross-sectional study involving 166 patients with type 2 diabetes.

## 2. Methods

### 2.1. Subjects

One hundred sixty-six patients (96 males and 70 females) with type 2 diabetes who were admitted to the Diabetes Center of the Osaka City University Hospital from 2012 to 2014 were recruited for this study. The diagnosis of diabetes was based on a previous history of diabetes or on criteria from the American Diabetes Association [21]. Patients with serum creatinine levels >1.5 mg/dL, acute and chronic inflammatory diseases, and malignant disease were excluded from this study. Each subject provided written informed consent. The ethical review board of our institution approved the study protocol (Registration Number 164).

Ten patients were treated with sulfonylureas monotherapy, 8 with dipeptidyl peptidase-4 (DPP-4) inhibitor monotherapy, 15 with biguanide (metformin) monotherapy, 2 with mitiglinide monotherapy, none with thiazolidine (pioglitazone) monotherapy, 61 were treated with a combination of these oral antidiabetic drugs, and 16 with diet therapy alone. Thirty-one patients were treated with insulin injections and 29 with a combination of oral antidiabetic drugs and insulin injections; 40.3% of subjects were treated with angiotensin II receptor blocker (ARB) and 47.6% of subjects were treated with statins.

### 2.2. Measurement of Soluble CD163 (sCD163) in the Blood

Peripheral serum samples were collected after 8 h overnight fasting. Then, serum sCD163 was determined by enzyme-linked immune sorbent assay (ELISA) (Quantikine® ELISA human CD163 immunoassay, R&D Systems, Inc., Minneapolis, MN, USA) as previously reported [22]. The intra-assay and interassay coefficients of variation of soluble CD163 were 3.5% and 4.1%, respectively.

### 2.3. Assessment of Monocyte Surface CD163 on Peripheral Blood Monocytes

For the assessment of cell surface CD163 levels on peripheral blood monocytes (monocyte CD163), 1 mL of peripheral whole blood was collected from each patient. Then, erythrocytes were lysed with lysing solution (lysing buffer, BD pharm Lyse™, BD Biosciences, San Diego, CA, USA) for 10 min; peripheral leukocytes were used in the assay. A fluorescent-conjugated monoclonal antibody against CD163 (phycoerythrin (PE) anti-human CD163 antibody, clone GHI/61, BioLegend Inc., San Diego, CA, USA) was used for labeling monocyte CD163. One hundred thousand-labeled leukocytes for each subject were measured by flow cytometry (BD FACSCanto™ flow cytometer, Becton Dickinson Biosciences, San Jose, CA, USA). Monocytes were first gated in a forward scatter and sideward scatter dot plot, and then the levels of CD163 mean florescent intensity (MFI) were measured within the monocyte gate.

### 2.4. Laboratory Measurements

Blood was drawn after an overnight fast; biochemical parameters were analyzed using standard laboratory methods as previously described [23]. Glycated hemoglobin A1C (HbA1c) levels were estimated as National Glycohemoglobin Standardization Program equivalent values (%), using the conversion formula established by the Japan Diabetes Society. Serum immune-reactive insulin (IRI) levels and serum C-peptide levels were measured by an electrochemiluminescence immunoassay (Roche Diagnostics K.K., Tokyo, Japan). High sensitivity TNF*α* (hsTNF*α*) and high sensitivity C-reactive proteins (hsCRP) were measured using a commercial ELISA kit (Quantikine HS ELISA human TNF*α* immunoassay and Quantikine ELISA human C-reactive protein immunoassay, R&D Systems, Inc., Minneapolis, MN, USA).

### 2.5. Assessment of Insulin Resistance by Homeostasis Model Assessment (HOMA-R)

HOMA-R was calculated from the fasting plasma glucose (FPG) and fasting plasma insulin (FIRI) levels according to the following formula previously reported by Matthews et al. [24]:

(1) $$\begin{array}{c} HOMA‐R=FIRI\text{ in }\mu U/mL\times FPG\text{ in }mmol/L/22.5. \end{array}$$

The HOMA-R index is a well-established insulin resistance index that is highly correlated with the insulin resistance index assessed by euglycemic hyperinsulinemic clamp, a gold standard technique for the evaluation of insulin resistance in type 2 diabetes [25–27].

### 2.6. Statistical Analysis

All values are the means ± SD or median (interquartile) as appropriate. Statistical analysis was performed using the JMP 10 (SAS Institute Inc., Cary, NC, USA). To analyze the relationships between monocyte CD163, sCD163, HOMA-R, and various clinical parameters, simple linear regression analyses or multiple regression analyses were performed as appropriate. Log transformations were performed to achieve a normal distribution and were utilized for regression analyses. *P* values of <0.05 were considered to be statistically significant.

## 3. Results

### 3.1. Clinical Characteristics of the Subjects

The clinical characteristics of all subjects are shown in Table 1. The mean age, duration of diabetes, and BMI were 61.0 ± 13.0 years, 13.6 ± 11.4 years, and 25.7 ± 5.5 kg/m^2^, respectively. Mean serum creatinine levels were 0.80 ± 0.23 mg/dL. The glycemic control was HbA1c 8.31 ± 1.66%. As an inflammatory cytokine, the median hsTNF*α* level was 1.57 pg/mL (interquartile ranged from 1.16–1.92 pg/mL), and the median hsCRP level was 604.3 ng/mL (interquartile ranged from 291.1–1681.5 ng/mL).

### 3.2. Serum sCD163 and Monocyte CD163 Levels

**Figure 1:**
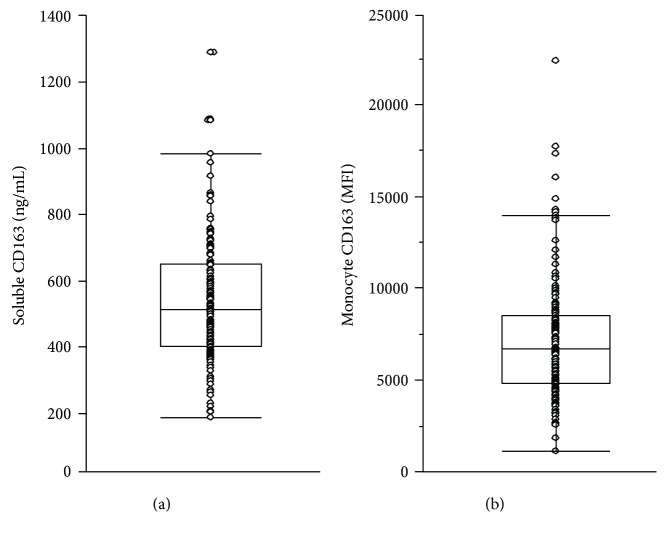
Level (ng/mL) of serum soluble CD163 (sCD163) (a) as measured by ELISA and (b) determination of monocyte surface CD163 level by flow cytometer (MFI, mean florescent intensity) in 166 patients with type 2 diabetes. The box denotes the median as well as the 10, 25, 75, and 90 percentiles.

The median serum sCD163 level was 582.9 ng/mL (interquartile ranged from 472.4 to 720.0 ng/mL), and the median monocyte CD163 levels were 6061 MFI (interquartile ranged from 4486 to 7876 MFI), as shown in Figures 1(a) and 1(b), respectively. The association between sCD163 and monocyte CD163 was not significant (*r* = 0.019, *p* = 0.808). Serum sCD163 levels were significantly correlated with hsTNF*α* (*r* = 0.204, *p* = 0.009). In contrast, monocyte CD163 levels showed a negative correlation trend with TNF*α* but were not statistically significant (*r* = –0.105, *p* = 0.186). Both sCD163 and monocyte CD163 levels were not associated with hsCRP (*r* = 0.065, *p* = 0.399; *r* = –0.110, *p* = 0.158, resp.).

### 3.3. Correlation between sCD163, Monocyte CD163, and Possible Clinical Factors Involved in Insulin Resistance Using HOMA-R

**Figure 2:**
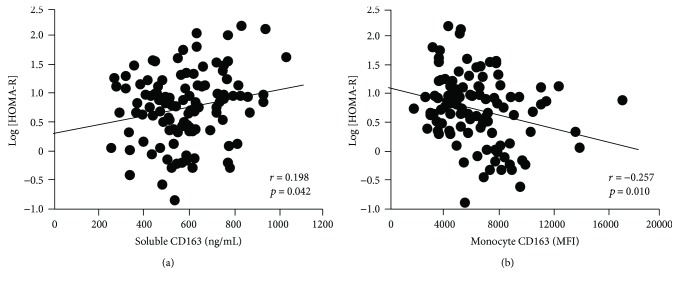
The association of soluble CD163 (sCD163) (a) and monocyte surface CD163 (b) using log-transformed HOMA-R as an insulin resistance index in 166 patients with type 2 diabetes. In a simple regression analysis, both the serum sCD163 and monocyte CD163 levels were significantly correlated with log-transformed HOMA-R (*r* = 0.198, *p* = 0.042; *r* = –0.257, *p* = 0.010, resp.).

The association between possible clinical factors and insulin resistance was analyzed by simple regression analyses as shown in Table 2. BMI (*r* = 0.392, *p* < 0.001), TG (*r* = 0.195, *p* = 0.043), HDL-C (*r* = –0.255, *p* = 0.008), hsTNF*α* (*r* = 0.200, *p* = 0.038), and hsCRP (*r* = 0.306, *p* = 0.001) were significantly associated with log-transformed HOMA-R values, respectively. Notably, sCD163 was significantly correlated with log [HOMA-R] (*r* = 0.198, *p* = 0.040), and monocyte CD163 levels were inversely correlated with log [HOMA-R] (*r* = –0.257, *p* = 0.010) (Figure 2).

### 3.4. Contributions of sCD163, Monocyte CD163, and Possible Clinical Factors Related to Insulin Resistance by HOMA-R and Multiple Regression Analysis

To explore the independent contribution of sCD163, monocyte CD163, and possible clinical factors to insulin resistance, multiple regression analyses were performed. Log [HOMA-R] was set as the dependent variable, and sCD163, monocyte CD163, and possible clinical factors such as age, sex, BMI, log [TG], HDL-C, Cre, log [hsTNF*α*], and log [hsCRP] were set as the independent variables as shown in Table 3. The monocyte CD163 (*β* = –0.220, *p* = 0.020) as well as the BMI (*β* = 0.277, *p* = 0.023) was found to be a statistically significant independent factor to log [HOMA-R] (*R*^2^ = 0.281, *p* = 0.001, as shown in Table 3). On the other hand, the significant contribution of sCD163 to log [HOMA-R] disappeared after adjusting for age, sex, BMI, and other factors (as in model 1 of Table 3).

## 4. Discussion

The present study demonstrated the inverse association of monocyte CD163 level in patients with type 2 diabetes having insulin resistance, even after adjusting for various clinical factors related to insulin resistance, as evaluated by HOMA-R in multivariate analysis as well as by univariate analysis. On the other hand, the serum sCD163 level was not significantly associated with insulin resistance in multivariate analysis after adjustment, despite a significant positive association with it in univariate analysis. Our findings provide the first demonstration of the stronger contribution of monocyte CD163 levels in the peripheral blood compared to the serum sCD163 level for insulin resistance in patients with type 2 diabetes, suggesting the possibility of its use as a novel clinical surrogate marker for insulin resistance and providing new insight into the role of CD163 in insulin resistance and inflammation.

Several previous studies have reported serum sCD163 levels in obesity and atherosclerotic disease and its association with insulin resistance [9–13]. The level of sCD163 was reported to be higher in subjects with obesity [10] and those with obese type 2 diabetes [11] compared to that in controls. Compared to the characteristics of the subjects in that study, our subjects were older in age, had lower BMI, and higher HbA1c levels. However, our data still showed the same trend of a significant association between sCD163 level and insulin resistance by HOMA-R. Taken together with those of the previous findings, the association between sCD163 and insulin resistance may be found in a wide range of glucose intolerance states, such as obese/impaired glucose tolerance for diabetic patients.

Regarding insulin resistance, the level of sCD163 was also reported to be associated with insulin resistance by HOMA-R in subjects with obesity and glucose intolerance [10, 12]. Of 95 healthy subjects, 65 were obese and 30 were normal weight. Zanni et al. revealed that sCD163 strongly correlated with HOMA-R (Spearman's *p* = 0.37, *p* = 0.0003) and that such an association remained significant even after adjustment for age, sex, visceral adiposity, and inflammatory markers [10]. Among the 234 participants, 96 had type 2 diabetes, 34 showed impaired glucose tolerance (IGT), and 104 had normal glucose tolerance (NGT). Groups were also matched for sex and BMI. Parkner et al demonstrated that sCD163 was independently associated with HOMA-R by both univariate analysis (*r* = 0.44) and multiple regression analysis [12]. Although our univariate analysis findings were also compatible with those studies, the association between sCD163 and insulin resistance is rather weak than monocyte CD163 in patients with type 2 diabetes. This is why such an association was not found after adjustment of possible clinical factors for insulin resistance in our multivariate analysis (Table 2). Interestingly, serum sCD163 levels were reported to predict the incidence of type 2 diabetes in a large prospective cohort study, in which 8849 general participants from Denmark were followed for 18 years [14]. Recently, in a double-blind randomized control trial of 72 patients with type 2 diabetic patients, intake of DHA-enriched fish oil for 8 weeks induced a significant decrease in the level of sCD163 accompanied by a change in insulin resistance-associated parameters [13]. These findings suggest that sCD163 is one of the biomarkers responsible for impaired glucose tolerance or insulin resistance induced by inflammation [14].

In our study, we focused on monocyte cell surface CD163 rather than sCD163, hypothesizing that monocyte surface CD163 might have a stronger anti-inflammatory effect. Currently, macrophages are categorized into two subsets, inflammatory macrophages (M1) with low CD163 expression and anti-inflammatory macrophages (M2) with high CD163 expression, which produce anti-inflammatory cytokines such as Interleukin-10 (IL-10) [28]. Similarly, monocytes, which are thought to be a precursor of macrophages, are also divided into anti-inflammatory and proinflammatory subsets by functional classification. Anti-inflammatory monocyte subsets are speculated to differentiate into M2 macrophages. Most upregulators of CD163 expression on monocyte have been reported to be anti-inflammatory cytokines such as IL-10 and glucocorticoids [20]. These recent findings suggest the possibly important role of monocyte CD163 in blood circulation. Previous studies measured CD163 mRNA levels in monocytes from the peripheral blood, which reflects the total transcription level of CD163 and, therefore, is not a direct estimate of the functional amount of CD163 [16, 17]. Based on these findings, we measured the expression of monocyte CD163 on the surface of monocytes by flow cytometry to more directly evaluate the functional amount of monocyte CD163 compared to that shown by CD163 mRNA or sCD163 levels.

As a result, monocyte surface CD163 was found to have a close inverse relationship with insulin resistance by HOMA-R, even after adjusting for other possible clinical factors related to insulin resistance, whereas sCD163 levels showed no association (Table 3). sCD163 is known to be produced from various tissue macrophages such as Kupffer cells, myeloid macrophages, and pulmonary macrophages, which indicates that sCD163 measurements reflect the total amount in the entire body. This may explain why sCD163 is a less sensitive marker for insulin resistance than monocyte CD163. Indeed, monocytes with low CD163 expression identified in our study can be considered to be a precursor of M1 macrophages located in local adipose tissue, which play an important role for insulin resistance [29]. Therefore, our data suggests that measurements of monocyte CD163, rather than sCD163, serve as a better surrogate marker for pathophysiological impact in insulin resistance for patients with type 2 diabetes.

The mechanisms underlying the role of monocyte CD163 in insulin resistance were not determined from our clinical study. However, other studies provide some clues. One possibility is that oxidative stress induced by hyperglycemia and various factors in diabetes and/or insulin resistance may cause shedding of surface monocyte CD163 by TACE/ADAM17 activation, resulting in an increase of CD163^low^ monocytes and, subsequently, an increase in preM1 and M1-like monocytes [30, 31]. This M1 predominant state in adipose tissue can lead to an increase in local inflammation and insulin resistance mediated by cytokines such as TNF*α*.

Another possibility is improved insulin resistance resulting from IL-10. The M2 macrophage, which is thought to differentiate from CD163^high^-expressed monocyte, produces IL-10 in local adipose tissue and skeletal muscles [28]. For in vivo mice experiments, IL-10 administration was reported to restore glucose uptake decreased by IL-6 administration in skeletal muscle to that of the control level [32], suggesting an important role for cytokine-induced glucose uptake in skeletal muscles. These findings raise the possibility that monocytes in the peripheral blood having high levels of CD163 expression induce expression and/or activate M2 macrophages in skeletal muscles, resulting in an increase in the local production of IL-10 and glucose uptake in skeletal muscles. Further studies are needed to elucidate the mechanisms underlying the relationship between monocyte CD163 and insulin resistance in obese and diabetes.

There are a few limitations in our study. First, our study is of cross-sectional design and is therefore not able to draw conclusive causal relationship between monocyte CD163 and insulin resistance. Second, insulin resistance was evaluated by the simple surrogate index of HOMA-R, which is not a direct quantitative index of insulin-mediated glucose uptake in skeletal muscles and/or adipose tissue. However, HOMA-R has been established as an excellent surrogate marker for insulin resistance and has been used by many investigators as a surrogate marker for insulin resistance in patients with both obesity and type 2 diabetes with various conditions [24–27, 33]. Multifaceted medical intervention was performed in our diabetic subjects, since they were recruited from inpatients participating in educational program for diabetes. Thus, we can eliminate the possibility of any influence by various medical interventions in our findings.

In conclusion, the present study demonstrated the closer association between surface monocyte CD163-expressing monocytes from the peripheral blood, rather than serum sCD163 levels, and insulin resistance, independent of known clinical factors in patients with type 2 diabetes. These findings suggest not only the possible use of monocyte CD163 as a novel surrogate marker for insulin resistance in diabetes and/or obesity but also provide insight into the pathophysiological role of monocyte CD163 in the development of insulin resistance states. In order to clinically clarify the causal relationship between monocyte CD163 and insulin resistance, interventional trials for insulin resistance will be needed, in which monocyte CD163 will be evaluated with some cytokines (e.g., IL-6, IL-10, and/or TNF*α*) after intervention such as body weight reduction, exercise, and/or drugs (e.g., thiazolidinedione). Furthermore, animal models with monocyte specific overexpression or knockdown of CD163, if made in the future, might elucidate basic mechanisms between monocyte CD163 and insulin resistance.

## Figures and Tables

**Table 1:** Clinical characteristics of 166 patients with type 2 diabetes.

*N* (male/female)	166 (96/70)
Age (years)	61.0 ± 13.0
Duration (years)	13.6 ± 11.4
BMI (kg/m^2^)	25.7 ± 5.48
SBP (mmHg)	128 ± 17.4
DBP (mmHg)	73.8 ± 9.3
HbA1c (%)	8.31 ± 1.66
HOMA-R	2.12 (1.22–3.05)
TC (mg/dL)	174 ± 51.2
TG (mg/dL)	110 (81–136)
LDL-C (mg/dL)	104 ± 36.6
HDL-C (mg/dL)	41.9 ± 11.9
FFA (mEq/L)	0.52 ± 0.23
Cre (mg/dL)	0.80 ± 0.23
ARB (yes/no)	67/99
Statin (yes/no)	79/87
hsTNF*α* (pg/mL)	1.57 (1.16–1.92)
hsCRP (ng/mL)	604.3 (291.1–1681.5)
Soluble CD163 (ng/mL)	582.9 (472.4–720.0)
Monocyte CD163 (MFI)	6061 (4486–7876)

Data are expressed as *n* (%), mean ± SD, or median (interquartile range) as appropriate. BMI: body mass index; SBP: systolic blood pressure; DBP: diastolic blood pressure; HbA1c: glycated hemoglobin A1c; HOMA-R: homeostasis model assessment of insulin resistance; TC: total cholesterol; TG: triglycerides; LDL-C: low-density lipoprotein cholesterol; HDL-C: high-density lipoprotein cholesterol; FFA: free fatty acid; Cre: creatinine; ARB: angiotensin-II receptor antagonists; hsTNF*α*: high sensitive tumor necrosis factor alpha; hsCRP: high sensitive C reactive protein.

**Table 2:** Correlation between insulin resistance by log-transformed HOMA-R values and possible clinical factors in 166 patients with type 2 diabetes by single regression analyses.

	Log [HOMA-R]	
	*r*	*p*
Age	−0.172	0.076
Duration	−0.146	0.139
BMI	0.392	<0.001
SBP	0.085	0.396
DBP	0.144	0.148
HbA1c	0.037	0.700
Log [TG]	0.195	0.043
LDL-C	0.114	0.241
HDL-C	−0.255	0.008
Log [FFA]	0.086	0.374
Cre	−0.002	0.981
Log [hsTNF*α*]	0.200	0.038
Log [hsCRP]	0.306	0.001
Soluble CD163	0.198	0.042
Monocyte CD163	−0.257	0.010

*r*: correlation coefficient by simple regression analysis. Log: log-transformed TG, FFA, hsTNF*α*, or hsCRP. The abbreviations are the same as shown in Table 1.

**Table 3:** Multiple regression analysis of sCD163, monocyte CD163, and possible clinical factors in insulin resistance by log-transformed HOMA-R values in 166 patients with type 2 diabetes.

	Model 1		Model 2		Model 3	
	*β*	*p*	*β*	*p*	*β*	*p*
Age	0.035	0.775	0.061	0.610	0.026	0.831
Sex (male = 1, female = 0)	0.074	0.505	0.071	0.525	0.065	0.558
BMI	0.308	0.011	0.304	0.012	0.277	0.023
HbA1c	0.063	0.514	0.084	0.381	0.064	0.506
Log [TG]	0.021	0.838	0.025	0.807	0.035	0.736
HDL-C	−0.157	0.120	−0.167	0.106	−0.174	0.090
Cre	0.043	0.704	0.026	0.821	0.024	0.835
Log [hsTNF*α*]	0.011	0.916	0.024	0.825	0.007	0.946
Log [hsCRP]	0.174	0.137	0.130	0.205	0.113	0.271
Soluble CD163	0.135	0.143	—	—	0.137	0.153
Monocyte CD163	—	—	−0.212	0.025	−0.220	0.020

*R* ^2^ (*p*)	0.236 (0.003)		0.264 (0.001)		0.281 (0.001)	

*β*: standard correlation coefficient by multiple regression analysis. *R*^2^: multiple coefficients of determination. Log: log-transformed TG, FFA, hsTNF*α*, or hsCRP. The abbreviations are the same as shown in Table 1.
